# Supplementary material for: Fundamental limits on nonequilibrium sensing
Source: Nat Commun. 2025 Nov 20;16:10227. doi: 10.1038/s41467-025-65058-7 (PMC12635388; doi:10.1038/s41467-025-65058-7)
Supplement: Supplementary file 1 — Supplementary Information [file 41467_2025_65058_MOESM1_ESM.pdf]

Supplementary Information: Fundamental limits on nonequilibrium sensing

Andreas Dechant[1] and Eric Lutz[2]
[1]Department of Physics #1, Graduate School of Science, Kyoto University, Kyoto 606-8502, Japan
[2]Institute for Theoretical Physics I, University of Stuttgart, D-70550 Stuttgart, Germany
(Dated: October 6, 2025)

The Supplementary Information contains details about (I) the derivation of the Harada-Sasa relation for interacting subsystems, (II) the analysis of response and fluctuations in linear systems and (III) a demonstration of enhanced sensing in a discrete system.

I. HARADA-SASA RELATION FOR SUBSYSTEMS

A. Setup and second law

We consider the overdamped Langevin equation for a system Z with configuration $\boldsymbol{z} \in \mathbb{R}^d$ [1]

$$\boldsymbol{\gamma}\dot{\boldsymbol{z}}(t) = \boldsymbol{f}(\boldsymbol{z}(t)) + \sqrt{2\boldsymbol{\gamma} k_\mathrm{B} T}\boldsymbol{\xi}(t). \tag{1}$$

Here, $\boldsymbol{f}(\boldsymbol{z})$ describes the forces (conservative and non-conservative external forces, as well as interaction forces) acting in the system, T is the temperature of the environment and $\boldsymbol{\gamma}$ is a positive definite $\mathbb{R}^d \times \mathbb{R}^d$ friction matrix. $\boldsymbol{\xi}(t) \in \mathbb{R}^d$ is a vector of mutually independent Gaussian white noises. Here and in the following we set the Boltzmann constant $k_\mathrm{B} = 1$ to simplify the notation. The steady-state probability density $p(\boldsymbol{z})$ of this system is determined by the Fokker-Planck equation

$$\boldsymbol{\nabla}_z^\mathrm{T}\big(\boldsymbol{\nu}(\boldsymbol{z})p(\boldsymbol{z})\big) = 0 \qquad \text{with} \qquad \boldsymbol{\nu}(\boldsymbol{z}) = \boldsymbol{\gamma}^{-1}\big(\boldsymbol{f}(\boldsymbol{z}) - T\boldsymbol{\nabla}_z \ln p(\boldsymbol{z})\big). \tag{2}$$

The local mean velocity $\boldsymbol{\nu}^Z(\boldsymbol{z})$ also determines the rate of entropy production in the steady state [2],

$$\sigma = \frac{1}{T}\left\langle \boldsymbol{\nu}^\mathrm{T}\boldsymbol{\gamma}\boldsymbol{\nu}\right\rangle, \tag{3}$$

where $\langle \ldots \rangle$ denotes an average with respect to the steady-state probability density. Note that the entropy production rate vanishes only when the local mean velocity vanishes, which implies the condition

$$\boldsymbol{f}(\boldsymbol{z}) = T\boldsymbol{\nabla}_z \ln p(\boldsymbol{z}) \equiv -\boldsymbol{\nabla}_z U(\boldsymbol{z}) \qquad \Rightarrow \qquad p(\boldsymbol{z}) = \frac{e^{-\frac{U(\boldsymbol{z})}{T}}}{\int d\boldsymbol{z}\ e^{-\frac{U(\boldsymbol{z})}{T}}}. \tag{4}$$

That is, the entropy production rate vanishes only for conservative forces, in which case the steady state is an equilibrium state with the corresponding Boltzmann-Gibbs density. We can derive an equivalent expression for the entropy production rate,

$$\sigma = \frac{1}{T}\left\langle \big(\boldsymbol{f} - T\boldsymbol{\nabla}_z \ln p\big)^\mathrm{T}\boldsymbol{\nu}\right\rangle = \frac{1}{T}\left\langle \boldsymbol{f}^\mathrm{T}\boldsymbol{\nu}\right\rangle. \tag{5}$$

Here, we replaced one occurrence of $\boldsymbol{\nu}(\boldsymbol{z})$ with Eq. (2), and used that $\langle \boldsymbol{\nabla}\psi\boldsymbol{\nu}\rangle = 0$ for any gradient field $\boldsymbol{\psi}(\boldsymbol{z})$ due to Eq. (2). We can further rewrite this in terms of the Stratonovich product $\circ$ as [2]

$$\left\langle \boldsymbol{f}^\mathrm{T}\boldsymbol{\nu}\right\rangle = \left\langle \boldsymbol{f}^\mathrm{T}(\boldsymbol{z}(t)) \circ \dot{\boldsymbol{z}}(t)\right\rangle = \dot{Q}_\mathrm{diss}. \tag{6}$$

where we identify the rate of heat $\dot{Q}_\mathrm{diss}$ dissipated into the environment with the rate at which work is being done on the system. Since the entropy production rate is by definition positive, we therefore find the second law of thermodynamics in the steady state

$$\dot{Q}_\mathrm{diss} = T\sigma \geq 0. \tag{7}$$

In other words, the system is constantly dissipating heat into the environment, except when it is in equilibrium.

B. Subsystems and information thermodynamics

We now divide the degrees of freedom into two subsets, $Z = X + Y$ with $\boldsymbol{z} = (\boldsymbol{x}, \boldsymbol{y})$, which we identify with two subsystems X and Y, for example, the sensor and the demon. We further assume that the friction matrix $\boldsymbol{\gamma}$ is block-diagonal

$$\boldsymbol{\gamma} = \begin{pmatrix} \boldsymbol{\gamma}^X & 0 \\ 0 & \boldsymbol{\gamma}^Y \end{pmatrix}. \tag{8}$$

Physically, this condition means that the noise acting on the two subsystems is uncorrelated; it is also referred to as bipartite condition [3]. Under this assumption, the entropy production rate decomposes into two positive contributions

$$\sigma = \sigma^X + \sigma^Y \qquad \text{with} \qquad \sigma^X = \frac{1}{T} \left\langle \boldsymbol{\nu}^{X,\mathrm{T}} \boldsymbol{\gamma}^X \boldsymbol{\nu}^X \right\rangle, \tag{9}$$

and similar for σ^Y, where $\boldsymbol{\nu}^X(\boldsymbol{z})$ denotes the components of the local mean velocity vector corresponding to subsystem X. Repeating the same calculation as for the overall system, we have

$$\sigma^X = \frac{1}{T} \left\langle \left(\boldsymbol{f}^X - T \boldsymbol{\nabla}_x \ln p^Z \right)^{\mathrm{T}} \boldsymbol{\nu}^X \right\rangle_{\mathrm{st}}, \tag{10}$$

where $\boldsymbol{f}^X(\boldsymbol{z})$ are the forces acting on subsystem X and we write $p(\boldsymbol{z}) = p^Z(\boldsymbol{z}) = p^{X+Y}(\boldsymbol{x}, \boldsymbol{y})$ to clarify that we mean the probability density of the compound system Z. However, since $\boldsymbol{\nabla}_x \ln p^Z(\boldsymbol{z})$ is generally not a gradient field with respect to $\boldsymbol{z}$ (there exists no function $\psi(\boldsymbol{x}, \boldsymbol{y})$ such that $(\boldsymbol{\nabla}_x, \boldsymbol{\nabla}_y)\psi(\boldsymbol{x}, \boldsymbol{y}) = (\boldsymbol{\nabla}_x \ln p^Z(\boldsymbol{x}, \boldsymbol{y}), 0)$) this term does vanish and we have

$$\sigma^X = \frac{1}{T} \left\langle \boldsymbol{f}^{X,\mathrm{T}}(\boldsymbol{z}(t)) \circ \dot{\boldsymbol{x}}(t) \right\rangle - l^X = \frac{\dot{Q}^X_{\mathrm{diss}}}{T} - l^X. \tag{11}$$

Here, we $\dot{Q}^X_{\mathrm{diss}}$ is the rate at subsystem X dissipates heat into the environment and the quantity l^X is the so-called learning rate [3]

$$l^X = \left\langle \boldsymbol{\nu}^{X,\mathrm{T}} \boldsymbol{\nabla}_x \ln p^Z \right\rangle_{\mathrm{st}}. \tag{12}$$

Instead of the second law Eq. (7), we now have

$$\dot{Q}^X_{\mathrm{diss}} - T l^X = T \sigma^X \geq 0. \tag{13}$$

In particular, if the learning rate l^X is sufficiently negative, we can have $\dot{Q}^X_{\mathrm{diss}} < 0$, that is, subsystem X continuously absorbs heat from the environment and converts it into work, in apparent violation of the second law of thermodynamics. However, since we have a similar relation for subsystem Y,

$$\dot{Q}^Y_{\mathrm{diss}} - T l^y = T \sigma^Y \geq 0, \tag{14}$$

and the relation $l^X = -l^Y$, the second law Eq. (7) is restored for the overall system. In other words, the apparent negative dissipation of subsystem X is always (over-)compensated by a positive dissipation of the other subsystem Y, ensuring that the overall dissipation remains positive. Let us briefly motivate the term learning rate. The mutual information between the subsystems X and Y is defined as

$$I^{X:Y} = \int d\boldsymbol{x} \int d\boldsymbol{y} \ \ln \left(\frac{p^{X+Y}(\boldsymbol{x}, \boldsymbol{y})}{p^X(\boldsymbol{x}) p^Y(\boldsymbol{y})} \right) p(\boldsymbol{x}, \boldsymbol{y}) = D_{\mathrm{KL}}(p^{X+Y} \| p^X p^Y), \tag{15}$$

where $p^X(\boldsymbol{x}) = \int d\boldsymbol{y} \ p^{X+Y}(\boldsymbol{x}, \boldsymbol{y})$ is the marginal probability density of subsystem X and $D_{\mathrm{KL}}(p, q)$ denotes the Kullback-Leibler divergence between two probability densities p and q. The mutual information is a positive measure of the correlations between X and Y; it vanishes only when the two subsystems are independent, $p^{X+Y}(\boldsymbol{x}, \boldsymbol{y}) = p^X(\boldsymbol{x}) p^Y(\boldsymbol{y})$. For a time-dependent system, we can decompose the change in mutual information into two contributions that can be attributed to the two subsystems

$$d_t I^{X:Y} = l^X + l^Y \qquad \text{with} \qquad l^X = \left\langle \boldsymbol{\nu}^{X,\mathrm{T}} \boldsymbol{\nabla}_x \ln p^{X+Y} \right\rangle + d_t S^X, \tag{16}$$

where $S^X = -\int d\boldsymbol{x} \ \ln(p^X(\boldsymbol{x})) p^X(\boldsymbol{x})$ is the differential entropy of system X. Therefore, the learning rate l^X quantifies the rate at which the correlations between X and Y increase due to subsystem X; it is the rate at which subsystem

X is acquiring information (learning) about subsystem Y. In the steady state, the time-derivative of the mutual information as well as of the differential entropy vanish, however, the individual learning rates remain non-zero. Using that $l^X = -l^Y$ in this case, we can also write Eq. (13) as

$$\dot{Q}^X_{\text{diss}} + Tl^Y \geq 0 \qquad \Rightarrow \qquad \dot{Q}^X_{\text{diss}} \geq -Tl^Y. \tag{17}$$

Thus, a negative rate of dissipation of subsystem X necessarily requires a positive learning rate of subsystem Y. In other words, by acquiring information about X, subsystem Y (the "demon") can allow X to apparently violate the second law of thermodynamics; at the cost of increasing its own dissipation.

C. Power spectral density

So far, we only considered the average behavior of the system described by Eq. (1). However, due to its inherent stochasticity, any observable $a(\boldsymbol{z}(t))$ measured in the system Z is subject to the fluctuations of $\boldsymbol{z}(t)$. One way of characterizing these fluctuations is in terms of the power-spectral density (PSD). This can be defined by considering the finite-time Fourier transform of the observable [1],

$$\hat{a}_\tau(\omega) = \int_0^\tau dt\ e^{i\omega t} a(\boldsymbol{z}(t)). \tag{18}$$

The PSD is then defined as the long-time limit of the fluctuations of $\hat{a}_\tau(\omega)$,

$$S_a(\omega) = \lim_{\tau\to\infty} \frac{1}{\tau}\Big(\big\langle |\hat{a}_\tau(\omega)|^2 \big\rangle - |\left\langle \hat{a}_\tau(\omega)\right\rangle|^2 \Big). \tag{19}$$

The Wiener-Khinchin theorem states that this is equal to the Fourier-transform of the steady-state autocorrelation of $a(\boldsymbol{z}(t))$,

$$S_a(\omega) = \int_{-\infty}^{\infty} dt\ e^{i\omega t}\ \Big(\langle a(\boldsymbol{z}(t)) a(\boldsymbol{z}(0))\rangle - \langle a\rangle^2 \Big) = 2\int_0^\infty dt\ \cos(\omega t)\Big(\langle a(\boldsymbol{z}(t)) a(\boldsymbol{z}(0))\rangle - \langle a\rangle^2 \Big). \tag{20}$$

Note that, since the autocorrelation is symmetric in time, the PSD is real and symmetric in ω; in fact, it is positive as is clear from its definition. Using Parseval's identity, we further have for the integral over all frequencies,

$$\frac{1}{\pi}\int_0^\infty d\omega\ S_a(\omega) = \left\langle a^2\right\rangle - \langle a\rangle^2 = \lim_{t\to 0}\Big(\langle a(\boldsymbol{z}(t)) a(\boldsymbol{z}(0))\rangle - \langle a\rangle^2 \Big), \tag{21}$$

that is, the steady-state fluctuations of $a(\boldsymbol{z})$. Generally, the frequency integral over the PSD is determined by the short-time behavior of the autocorrelation function. We can similarly define the cross-PSD for two different observables $a(\boldsymbol{z}(t))$ and $b(\boldsymbol{z}(t))$,

$$S_{a,b}(\omega) = \int_0^\infty dt\ \cos(\omega t)\Big(\langle a(\boldsymbol{z}(t)) b(\boldsymbol{z}(0))\rangle + \langle b(\boldsymbol{z}(t)) a(\boldsymbol{z}(0)\rangle - 2\langle a\rangle \langle b\rangle \Big). \tag{22}$$

With the identification $S_a(\omega) = S_{a,a}(\omega)$, the PSD matrix

$$\boldsymbol{S}_{a,b}(\omega) = \begin{pmatrix} S_{a,a}(\omega) & S_{a,b}(\omega) \\ S_{a,b}(\omega) & S_{b,b}(\omega) \end{pmatrix} \tag{23}$$

is a positive definite matrix, whose frequency integral is equal to the steady-state covariance matrix of $a(\boldsymbol{z})$ and $b(\boldsymbol{z})$. This can be extended to an arbitrary number of observables, in particular, choosing the entries of $\boldsymbol{z}$ itself as observables, we can define the PSD matrix of $\boldsymbol{z}$,

$$\big(\boldsymbol{S}^Z(\omega)\big)_{kl} = S_{z_k,z_l}(\omega). \tag{24}$$

Recalling the decomposition into subsystems $Z = X + Y$, we can also write this as

$$\boldsymbol{S}^Z(\omega) = \begin{pmatrix} \boldsymbol{S}^X(\omega) & \boldsymbol{S}^{XY}(\omega) \\ \boldsymbol{S}^{XY,\mathrm{T}}(\omega) & \boldsymbol{S}^Y(\omega) \end{pmatrix}, \tag{25}$$

where $\boldsymbol{S}^X(\omega)$ is the PSD matrix of subsystem X. We remark that we can also define the corresponding PSD matrix for the velocity, i. e.

$$\big(\boldsymbol{S}_v^Z(\omega)\big)_{kl} = \int_0^\infty dt\ \cos(\omega t)\ \bigg(\Big(\langle \dot{z}_k(t)\dot{z}_l(0)\rangle + \langle \dot{z}_l(t)\dot{z}_k(0)\rangle\Big) - 2\langle \dot{z}_k\rangle\langle \dot{z}_l\rangle\bigg). \tag{26}$$

This is related to the PSD matrix of Z via

$$\boldsymbol{S}_v^Z(\omega) = \omega^2 \boldsymbol{S}^Z(\omega). \tag{27}$$

D. Response and Harada-Sasa relation

We now consider applying a small time-dependent perturbation $\epsilon\boldsymbol{\phi}(t)$ to the system described by Eq. (1),

$$\boldsymbol{\gamma}\dot{\boldsymbol{z}}(t) = \boldsymbol{f}(\boldsymbol{z}(t)) + \epsilon\boldsymbol{\phi}(t) + \sqrt{2\boldsymbol{\gamma}T}\boldsymbol{\xi}(t) \tag{28}$$

where $\epsilon \ll 1$ is a small parameter. We assume that we have $\boldsymbol{\phi}(t) = 0$ for $t < 0$ and that the system is initially in the steady state. We want to characterize the response of the system to this perturbation, which we describe in terms of the difference

$$\langle \boldsymbol{z}(t)\rangle^\epsilon - \langle \boldsymbol{z}\rangle, \tag{29}$$

that is, how much the expected value of $\boldsymbol{z}$ at time t changes relative to its steady-state value as a result of the perturbation. In the linear response regime, $\epsilon \to 0$, we expect this change to be proportional to ϵ and write it as

$$\langle \boldsymbol{z}(t)\rangle^\epsilon - \langle \boldsymbol{z}\rangle = \int_0^t dt'\ \langle \dot{\boldsymbol{z}}(t')\rangle^\epsilon \simeq \epsilon \int_0^t dt' \int_0^{t'} dt''\ \boldsymbol{\mathcal{R}}_v^Z(t'-t'')\boldsymbol{\phi}(t'') + O(\epsilon^2). \tag{30}$$

The matrix $\boldsymbol{\mathcal{R}}_v^Z(t'-t'')$ is the velocity response matrix, which describes the change in the velocity at time t' as a consequence of the applied perturbation force at time $t'' < t'$; specifically, $\mathcal{R}_{v,kl}^Z$ is the change of the velocity in direction k to an applied force in direction l. In general, we can express the response of an observable to the perturbation as a correlation function between the observable and some other quantity in the unperturbed system (see Refs. [4, 5]),

$$\langle \boldsymbol{z}(t)\rangle^\epsilon - \langle \boldsymbol{z}\rangle^0 \simeq \epsilon\langle \boldsymbol{z}(t)Q(t)\rangle^0, \tag{31}$$

where $\langle\ldots\rangle^0$ means the expectation with respect to the unperturbed dynamics. The quantity $Q(t)$ is the derivative of the path-probability density with respect to ϵ,

$$Q(t) = \partial_\epsilon \ln \mathbb{P}^\epsilon(\hat{\boldsymbol{z}})\Big|_{\epsilon=0} = \frac{1}{2T}\int_0^t dt''\ \boldsymbol{\phi}^{\mathrm{T}}(t'')\big(\dot{\boldsymbol{z}}(t'') - \boldsymbol{\gamma}^{-1}\boldsymbol{f}(\boldsymbol{z}(t''))\big) = \frac{1}{2T}\int_0^t dt''\ \boldsymbol{\phi}^{\mathrm{T}}(t'')\sqrt{2T\boldsymbol{\gamma}^{-1}}\boldsymbol{\xi}(t''), \tag{32}$$

where we used Eq. (1). We find

$$\langle \boldsymbol{z}(t)\rangle^\epsilon - \langle \boldsymbol{z}\rangle = \frac{1}{2T}\int_0^t dt' \int_0^t dt''\ \Big\langle \dot{\boldsymbol{z}}(t')\Big(\boldsymbol{\phi}^{\mathrm{T}}(t'')\sqrt{2T\boldsymbol{\gamma}^{-1}}\boldsymbol{\xi}(t'')\Big)\Big\rangle \tag{33}$$

Since the noise at later times $t'' > t'$ is independent of the velocity at time t', this part of the integral does not contribute, and we have

$$\langle \boldsymbol{z}(t)\rangle^\epsilon - \langle \boldsymbol{z}\rangle = \frac{1}{2T}\int_0^t dt' \int_0^{t'} dt''\ \Big\langle \dot{\boldsymbol{z}}(t')\Big(\boldsymbol{\phi}^{\mathrm{T}}(t'')\big(\dot{\boldsymbol{z}}(t'') - \boldsymbol{\gamma}^{-1}\boldsymbol{f}(\boldsymbol{z}(t''))\big)\Big)\Big\rangle, \tag{34}$$

which reflects causality, i. e. the perturbation only affects the velocity at a later time. Comparing this to Eq. (30), we can identify the response matrix

$$\boldsymbol{\mathcal{R}}_v^Z(t'-t'') = \frac{1}{2T}\Big\langle \dot{\boldsymbol{z}}(t')\big(\dot{\boldsymbol{z}}(t'') - \boldsymbol{\gamma}^{-1}\boldsymbol{f}(\boldsymbol{z}(t''))\big)^{\mathrm{T}}\Big\rangle \tag{35}$$

The first term is precisely the velocity correlation function, that is, we have for $t' \geq t''$,

$$\boldsymbol{\mathcal{R}}_v^Z(t'-t'') = \frac{1}{2T}\Big(\langle \dot{\boldsymbol{z}}(t')\dot{\boldsymbol{z}}^{\mathrm{T}}(t'')\rangle - \langle \dot{\boldsymbol{z}}(t')\boldsymbol{f}^{\mathrm{T}}(\boldsymbol{z}(t''))\rangle\,\boldsymbol{\gamma}^{-1}\Big). \tag{36}$$

Let us now consider the following expression

$$\mathrm{tr}\bigg(\boldsymbol{\gamma}\Big(\left\langle \dot{\boldsymbol{z}}(t)\dot{\boldsymbol{z}}^{\mathrm{T}}(0)\right\rangle - 2T\boldsymbol{\mathcal{R}}_v^Z(t)\Big)\bigg), \tag{37}$$

where tr denotes the trace. Plugging in the above expression for the response matrix, we obtain

$$\mathrm{tr}\bigg(\boldsymbol{\gamma}\Big(\left\langle \dot{\boldsymbol{z}}(t)\dot{\boldsymbol{z}}^{\mathrm{T}}(0)\right\rangle - 2T\boldsymbol{\mathcal{R}}_v^Z(t)\Big)\bigg) = \mathrm{tr}\bigg(\boldsymbol{\gamma}\left\langle \dot{\boldsymbol{z}}(t)\boldsymbol{f}^{\mathrm{T}}(\boldsymbol{z}(0))\right\rangle\boldsymbol{\gamma}^{-1}\bigg) = \left\langle \boldsymbol{f}^{\mathrm{T}}(\boldsymbol{z}(0))\dot{\boldsymbol{z}}(t)\right\rangle, \tag{38}$$

where we used the invariance of the trace under cyclic permutations. Multiplying by $\cos(\omega t)$ and integrating over t, we get

$$\mathrm{tr}\bigg(\boldsymbol{\gamma}\Big(\boldsymbol{S}_v^z(\omega) - 2T\boldsymbol{R}_v^Z(\omega)\Big)\bigg) = \int_0^\infty dt\ \cos(\omega t)\left\langle \boldsymbol{f}^{\mathrm{T}}(\boldsymbol{z}(0))\dot{\boldsymbol{z}}(t)\right\rangle, \tag{39}$$

where we identified the velocity PSD matrix Eq. (26). We also have for the the Fourier transform of the response matrix,

$$\boldsymbol{R}_v^Z(\omega) + i\tilde{\boldsymbol{R}}_v^Z(\omega) = \int_{-\infty}^\infty dt\ e^{i\omega t}\boldsymbol{\mathcal{R}}_v^Z(t) = \int_0^\infty dt\ e^{i\omega t}\boldsymbol{\mathcal{R}}_v^Z(t) = \int_0^\infty dt\ \cos(\omega t)\boldsymbol{\mathcal{R}}_v^Z(t) + i\int_0^\infty dt\ \sin(\omega t)\boldsymbol{\mathcal{R}}_v^Z(t), \tag{40}$$

since the response function vanishes for negative arguments due to causality. Finally, using that the integral over all frequencies is is equal to the short-time behavior of the integrand, or, more formally, the relation

$$\int_0^\infty d\omega\ \cos(\omega t) = \pi\delta(t), \tag{41}$$

we obtain the identity

$$\frac{1}{\pi}\int_0^\infty d\omega\ \mathrm{tr}\bigg(\boldsymbol{\gamma}\Big(\boldsymbol{S}_v^Z(\omega) - 2T\boldsymbol{R}_v^Z(\omega)\Big)\bigg) = \left\langle \boldsymbol{f}^{\mathrm{T}} \circ \dot{\boldsymbol{z}}\right\rangle = \dot{Q}_{\mathrm{diss}}. \tag{42}$$

This is known as the Harada-Sasa relation [5, 6]; it expresses the dissipated heat as an integral over the violation of the fluctuation-dissipation theorem. Since the friction matrix is assumed as block-diagonal, we can decompose the expression in Eq. (37) into contributions due to X and Y,

$$\begin{aligned}\mathrm{tr}\bigg(\boldsymbol{\gamma}\Big(\left\langle \dot{\boldsymbol{z}}(t)\dot{\boldsymbol{z}}^{\mathrm{T}}(0)\right\rangle - 2T\boldsymbol{\mathcal{R}}_v^Z(t)\Big)\bigg) &= \mathrm{tr}\bigg(\boldsymbol{\gamma}^X\Big(\left\langle \dot{\boldsymbol{x}}(t)\dot{\boldsymbol{x}}^{\mathrm{T}}(0)\right\rangle - 2T\boldsymbol{\mathcal{R}}_v^X(t)\Big)\bigg) + \mathrm{tr}\bigg(\boldsymbol{\gamma}^Y\Big(\left\langle \dot{\boldsymbol{y}}(t)\dot{\boldsymbol{y}}^{\mathrm{T}}(0)\right\rangle - 2T\boldsymbol{\mathcal{R}}_v^y(t)\Big)\bigg)\\ &= \left\langle \boldsymbol{f}^{X,\mathrm{T}}(0)\dot{\boldsymbol{x}}(t)\right\rangle + \left\langle \boldsymbol{f}^{Y,\mathrm{T}}(0)\dot{\boldsymbol{y}}(t)\right\rangle,\end{aligned} \tag{43}$$

where we write the response matrix in block form similar to the PSD matrix in Eq. (25),

$$\boldsymbol{\mathcal{R}}^Z(t) = \begin{pmatrix} \boldsymbol{\mathcal{R}}^X(t) & \boldsymbol{\mathcal{R}}^{XY}(t) \\ \boldsymbol{\mathcal{R}}^{YX}(t) & \boldsymbol{\mathcal{R}}^Y(t) \end{pmatrix}. \tag{44}$$

The matrix $\boldsymbol{\mathcal{R}}^X(t)$ describes the response of subsystem X to a perturbation applied to X, while $\boldsymbol{\mathcal{R}}^{XY}(t)$ describes the response of X to a perturbation applied to Y. Note that, in contrast to the PSD matrix the response matrix is generally not symmetric, $\boldsymbol{\mathcal{R}}^{YX}(t) \neq \boldsymbol{\mathcal{R}}^{XY,\mathrm{T}}(t)$. Repeating the same calculation as above, we therefore find for subsystem X,

$$\frac{1}{\pi}\int_0^\infty d\omega\ \mathrm{tr}\bigg(\boldsymbol{\gamma}^X\Big(\boldsymbol{S}_v^X(\omega) - 2T\boldsymbol{R}_v^X(\omega)\Big)\bigg) = \left\langle \boldsymbol{f}^{X,\mathrm{T}} \circ \dot{\boldsymbol{x}}\right\rangle = \dot{Q}^X_{\mathrm{diss}}. \tag{45}$$

Thus, the Harada-Sasa relation also holds separately for each subsystem—the violation of the fluctuation-dissipation theorem of subsystem X is equal to the heat dissipated by subsystem X. As discussed before, the latter can be negative under suitable conditions, which implies that, compared to an equilibrium system, the response can be enhanced relative to the fluctuations.

We remark that in equilibrium we have the fluctuation-dissipation theorem

$$\boldsymbol{S}_v^Z(\omega) = 2T\boldsymbol{R}_v^Z(\omega), \tag{46}$$

which relates the power spectral density to the real part of the velocity response function. Out of equilibrium, we can define a frequency-dependent effective temperature [7]

$$T^Z_{\text{eff}}(\omega) = \frac{\text{tr}\big(\boldsymbol{\gamma}\boldsymbol{S}^Z_v(\omega)\big)}{2\text{tr}\big(\boldsymbol{\gamma}\boldsymbol{R}^Z_v(\omega)\big)}, \tag{47}$$

which quantifies the deviations from the fluctuation-dissipation theorem and is equal to the environmental temperature in equilibrium. We can apply similar definitions to the subsystems X and Y; out of equilibrium, the effective temperatures of the subsystems can be different from the overall one. In particular, if the effective temperature of a subsystem is lower than the environmental temperature, then this implies that the response at a given frequency is enhanced compared to the fluctuations at the same frequency.

II. RESPONSE AND FLUCTUATIONS FOR LINEAR SYSTEMS

A. General relations

Let us now consider a particular case of Eq. (1) with a linear force

$$\boldsymbol{\gamma}\dot{\boldsymbol{z}}(t) = -\boldsymbol{K}\boldsymbol{z}(t) + \sqrt{2\boldsymbol{\gamma}T}\boldsymbol{\xi}(t). \tag{48}$$

Here, the force matrix $\boldsymbol{K}$ is assumed to have eigenvalues with strictly positive real parts, so that the system has a stable steady state. In this case, we can determine the steady-state explicitly; it is given by the Gaussian probability density

$$p^Z_{\text{st}}(\boldsymbol{z}) = \frac{1}{(2\pi)^d \det(\boldsymbol{\Xi}^Z)} \exp\bigg(- \frac{1}{2}\boldsymbol{z}^{\text{T}}\boldsymbol{\Xi}^{Z^{-1}}\boldsymbol{z}\bigg), \tag{49}$$

whose covariance matrix is the solution of the Lyapunov equation

$$\boldsymbol{\gamma}^{-1}\boldsymbol{K}\boldsymbol{\Xi}^Z + \boldsymbol{\Xi}^Z\boldsymbol{K}^{\text{T}}\boldsymbol{\gamma}^{-1} = 2T\boldsymbol{\gamma}^{-1}. \tag{50}$$

To keep the notation compact, we define the matrices $\boldsymbol{A} = \boldsymbol{\gamma}^{-1}\boldsymbol{K}$ and $\boldsymbol{B} = \boldsymbol{\gamma}^{-1}T$, so that

$$\boldsymbol{A}\boldsymbol{\Xi}^Z + \boldsymbol{\Xi}^Z\boldsymbol{A}^{\text{T}} = 2\boldsymbol{B}. \tag{51}$$

The local mean velocity is given by

$$\boldsymbol{\nu}(\boldsymbol{z}) = \big(- \boldsymbol{A} + \boldsymbol{B}\boldsymbol{\Xi}^{Z^{-1}}\big)\boldsymbol{z}, \tag{52}$$

and the entropy production rate by

$$\sigma = \text{tr}\Big(\boldsymbol{B}^{-1}\big(- \boldsymbol{A} + \boldsymbol{B}\boldsymbol{\Xi}^{Z^{-1}}\big)\boldsymbol{\Xi}^Z\big(- \boldsymbol{A} + \boldsymbol{B}\boldsymbol{\Xi}^{Z^{-1}}\big)^{\text{T}}\Big) = \frac{1}{T}\text{tr}\Big(\boldsymbol{\gamma}^{-1}\big(\boldsymbol{K}\boldsymbol{\Xi}^Z\boldsymbol{K}^{\text{T}} - \boldsymbol{K}\big)\Big), \tag{53}$$

where we used Eq. (50) in the second equality. The system is in equilibrium if the the force matrix $\boldsymbol{K}$ is symmetric, in which case we have $\boldsymbol{\Xi}^Z = T\boldsymbol{K}^{-1}$ and thus $\boldsymbol{A} = \boldsymbol{B}\boldsymbol{\Xi}^{Z^{-1}}$ and $\sigma = 0$. The average of $\boldsymbol{z}$ at time t, conditioned on a value $\boldsymbol{z}'$ at time 0 is given by

$$\langle \boldsymbol{z}(t) | \boldsymbol{z}' \rangle = e^{-\boldsymbol{A}t}\boldsymbol{z}', \tag{54}$$

which allows us to compute the correlation function

$$\big\langle \boldsymbol{z}(t)\boldsymbol{z}^{\text{T}}(0)\big\rangle = \Big\langle \langle \boldsymbol{z}(t)|\boldsymbol{z}'\rangle\, \boldsymbol{z}'^{\text{T}} \Big\rangle = e^{-\boldsymbol{A}t}\boldsymbol{\Xi}^Z. \tag{55}$$

Using this, we can evaluate the velocity PSD matrix

$$\boldsymbol{S}^Z_v(\omega) = \omega^2 \int_0^\infty dt\ \cos(\omega t)\Big(e^{-\boldsymbol{A}t}\boldsymbol{\Xi}^Z + \boldsymbol{\Xi}^Z e^{-\boldsymbol{A}^{\text{T}}t}\Big) = \omega^2\Big(\big(\boldsymbol{A}^2 + \omega^2\boldsymbol{I}\big)^{-1}\boldsymbol{A}\boldsymbol{\Xi}^Z + \boldsymbol{\Xi}^Z\boldsymbol{A}^{\text{T}}\big(\boldsymbol{A}^2 + \omega^2\boldsymbol{I}\big)^{-1,\text{T}}\Big), \tag{56}$$

and the Fourier-transformed velocity response matrix is given by

$$\boldsymbol{R}_v^Z(\omega) + i\tilde{\boldsymbol{R}}_v^Z(\omega) = \boldsymbol{\gamma}^{-1}\big(\omega^2\boldsymbol{I} + i\omega\boldsymbol{A}\big)\big(\boldsymbol{A}^2 + \omega^2\boldsymbol{I}\big)^{-1}, \tag{57}$$

where $\boldsymbol{I}$ denotes the identity matrix.

As in Sec. I B, we now decompose the system into two subsystems, $Z = X + Y$ with $\boldsymbol{z} = (\boldsymbol{x}, \boldsymbol{y})$, where we interpret X as the sensor and Y as an auxiliary system ("demon") that we engineer to enhance the performance of X. From Eq. (12), we can write the learning rate of subsystem X as

$$l^X = \big\langle \boldsymbol{\nu}^{X,\mathrm{T}}\boldsymbol{\nabla}_x \ln p^Z\big\rangle = -\big\langle \boldsymbol{\nabla}_x^{\mathrm{T}}\boldsymbol{\nu}^X\big\rangle = \mathrm{tr}\Big(\big(\boldsymbol{A} - \boldsymbol{B}\boldsymbol{\Xi}^{Z^{-1}}\big)^X\Big) = \mathrm{tr}\Big(\boldsymbol{\gamma}^{X^{-1}}\Big(\boldsymbol{K}^X - T\big(\boldsymbol{\Xi}^{Z^{-1}}\big)^X\Big)\Big), \tag{58}$$

where $\boldsymbol{K}^X$ denotes the upper left block of the matrix $\boldsymbol{K}$, corresponding to subsystem X. Note that this expression involves the upper-left block of the inverse covariance matrix of the compound system, which includes correlations between X and Y. Specifically, using the block-inversion formula for matrices, we have

$$\big(\boldsymbol{\Xi}^{Z^{-1}}\big)^X = \big(\boldsymbol{\Xi}^X - \boldsymbol{\Xi}^{XY}\boldsymbol{\Xi}^{Y^{-1}}\boldsymbol{\Xi}^{XY^{\mathrm{T}}}\big)^{-1}. \tag{59}$$

The entropy production rate of X is given by

$$\sigma^X = \mathrm{tr}\left(\boldsymbol{\gamma}^{X^{-1}}\left(\frac{1}{T}\boldsymbol{K}\boldsymbol{\Xi}^Z\boldsymbol{K}^{\mathrm{T}} - 2\boldsymbol{K} + T\boldsymbol{\Xi}^{Z^{-1}}\right)^X\right) \tag{60}$$

and the dissipation rate by

$$\dot{Q}_{\mathrm{diss}}^X = T\big(\sigma^X + l^X\big) = \mathrm{tr}\Big(\boldsymbol{\gamma}^{X^{-1}}\big(\boldsymbol{K}\boldsymbol{\Xi}^Z\boldsymbol{K}^{\mathrm{T}} - T\boldsymbol{K}\big)^X\Big). \tag{61}$$

B. Solvable two-dimensional model

While the above relations hold for general linear dynamics, an explicit evaluation requires solving the Lyapunov equation Eq. (50). We therefore focus on the two-dimensional case, where this can be done explicitly. Specifically, we set

$$\boldsymbol{K} = \begin{pmatrix} k_x + \kappa & -\kappa - \delta \\ -\kappa + \delta & k_y + \kappa \end{pmatrix}, \qquad \boldsymbol{\gamma} = \gamma\boldsymbol{I}. \tag{62}$$

This describes two linearly interacting overdamped degrees of freedom x and y. k_x and k_y are the corresponding one-body force constants, κ describes a reciprocal interaction and δ a non-reciprocal interaction. The system is out of equilibrium if $\delta \neq 0$. We define the constants

$$\mathcal{T} = \mathrm{tr}(\boldsymbol{K}) = k_x + k_y + 2\kappa, \qquad \mathcal{D} = \det(\boldsymbol{K}) = k_x k_y + (k_x + k_y)\kappa + \delta^2, \qquad \mathcal{Q} = k_y + \kappa. \tag{63}$$

In terms of these, the eigenvalues of the force matrix $\boldsymbol{K}$ can be written as

$$\lambda^{\pm} = \frac{1}{2}\Big(\mathcal{T} \pm \sqrt{\mathcal{T}^2 - 4\mathcal{D}}\Big) \tag{64}$$

The requirement for a stable steady state thus implies $\mathcal{T} > 0$ and $\mathcal{D} > 0$. The resulting covariance matrix is given by

$$\boldsymbol{\Xi} = \frac{T}{\mathcal{T}\mathcal{D}}\begin{pmatrix} k_y^2 + 3k_y\kappa + k_x(k_y + \kappa) + 2(\delta^2 + \delta\kappa + \kappa^2) & k_x(\kappa + \delta) + k_y(\kappa - \delta) + 2\kappa^2 \\ k_x(\kappa + \delta) + k_y(\kappa - \delta) + 2\kappa^2 & k_x^2 + 3k_y\kappa + k_y(k_x + \kappa) + 2(\delta^2 - \delta\kappa + \kappa^2) \end{pmatrix}. \tag{65}$$

Using this, we can write the dissipation rate of subsystem X, its velocity-PSD and velocity-response as

$$\dot{Q}_{\mathrm{diss}}^X = \frac{2T\delta(\delta + \kappa)}{\gamma\mathcal{T}}, \qquad S_v^X(\omega) = \frac{2T}{\gamma}\frac{(\gamma\omega)^2\big(\mathcal{Q}^2 + (\delta + \kappa)^2 + (\gamma\omega)^2\big)}{\mathcal{D}^2 + \big(\mathcal{T}^2 - 2\mathcal{D}\big)(\gamma\omega)^2 + (\gamma\omega)^4} \tag{66}$$

$$R_v^X(\omega) = \frac{1}{\gamma}\frac{(\gamma\omega)^2\big(\mathcal{Q}^2 + \kappa^2 - \delta^2 + (\gamma\omega)^2\big)}{\mathcal{D}^2 + \big(\mathcal{T}^2 - 2\mathcal{D}\big)(\gamma\omega)^2 + (\gamma\omega)^4} \qquad \tilde{R}_v^X(\omega) = \frac{1}{\gamma}\frac{(\gamma\omega)\big(\mathcal{Q}\mathcal{D} + (k_x + \kappa)(\gamma\omega)^2\big)}{\mathcal{D}^2 + \big(\mathcal{T}^2 - 2\mathcal{D}\big)(\gamma\omega)^2 + (\gamma\omega)^4}.$$

In equilibrium, that is, for $\delta = 0$, we verify the fluctuation-dissipation theorem,

$$S^X_{v,\mathrm{eq}}(\omega) = 2TR^X_{v,\mathrm{eq}}(\omega). \tag{67}$$

Out of equilibrium, the ratio of the PSD and real part of the response can be used to calculate the effective temperature Eq. (47)

$$\frac{T^X_{\mathrm{eff}}(\omega)}{T} = \frac{S^X_v(\omega)}{2TR^X_v(\omega)} = \frac{\mathcal{Q}^2 + (\delta+\kappa)^2 + (\gamma\omega)^2}{\mathcal{Q}^2 + \kappa^2 - \delta^2 + (\gamma\omega)^2} = 1 + \frac{2\delta(\delta+\kappa)}{\mathcal{Q}^2 + \kappa^2 - \delta^2 + (\gamma\omega)^2} = 1 + \frac{\gamma\mathcal{T}\dot{Q}^X_{\mathrm{diss}}}{T\big(\mathcal{Q}^2 + \kappa^2 - \delta^2 + (\gamma\omega)^2\big)}. \tag{68}$$

This relation implies that, as expected from Eq. (45), an effective temperature that is lower than the environmental temperature (and thus enhanced response relative to the fluctuations) is only possible when $\dot{Q}^X_{\mathrm{diss}}$ is negative. From Eq. (17), this also implies that the subsystem Y has to continuously acquire information about X in order to facilitate the reduction in effective temperature.

C. Optimization of the signal-to-noise ratio

As in the main text, we introduce the signal-to-noise ratio (SNR) of system X,

$$\mathrm{SNR}^X(\omega) = \frac{\bar{R}^X_v(\omega) f}{\omega\sqrt{\Xi^X}}. \tag{69}$$

Here, $\bar{R}^X_v(\omega) = \sqrt{R^X_v(\omega)^2 + \tilde{R}^X_v(\omega)^2}$ is the magnitude of the velocity response, which is related to the response of the coordinate x as $\bar{R}^X_v(\omega) = \omega\bar{R}^X(\omega)$. f is the magnitude of the perturbation force applied to X and $\Xi^X = \mathrm{Var}_{\mathrm{st}}(x)$ denotes the steady-state fluctuations of x. Our goal is to maximize the SNR maintaining the magnitude of the response. We rewrite the response function and fluctuations using the eigenvalues $\lambda^\pm$ of the force matrix, as well as the entropy production rate σ,

$$\bar{R}^X_v(\omega)^2 = \frac{\omega^2\big(\mathcal{Q}^2 + (\gamma\omega)^2\big)}{\big((\lambda^+)^2 + (\gamma\omega)^2\big)\big((\lambda^-)^2 + (\gamma\omega)^2\big)}, \qquad \Xi^X = \frac{T(\gamma\sigma + 2\mathcal{Q}) - \sqrt{\gamma\sigma\big(\gamma\sigma - 4\frac{(\mathcal{Q}-\lambda^+)(\mathcal{Q}-\lambda^-)}{\lambda^+ + \lambda^-}\big)}}{2\lambda^+\lambda^-}. \tag{70}$$

We see that fixing the response function for all frequencies also determines the parameters $\lambda^\pm$ and $\mathcal{Q}$, while the entropy production rate σ only enters the fluctuations. Maximizing the SNR at a given response therefore corresponds to minimizing Ξ^X with respect to σ. The minimal value is attained in the limit $\sigma \to \infty$, that is, when driving the system far from equilibrium. We find

$$\frac{\Xi^X_{\mathrm{min}}}{\Xi^X_{\mathrm{eq}}} = \frac{k_{x,\mathrm{eq}} + \frac{k_{y,\mathrm{eq}}\kappa_{\mathrm{eq}}}{k_{y,\mathrm{eq}} + \kappa_{\mathrm{eq}}} + k_{y,\mathrm{eq}} + \kappa_{\mathrm{eq}}}{k_{x,\mathrm{eq}} + k_{y,\mathrm{eq}} + 2\kappa_{\mathrm{eq}}} < 1. \tag{71}$$

That is, for a given equilibrium system with parameters $k_{x,\mathrm{eq}}$, $k_{y,\mathrm{eq}}$ and κ_{eq}, we can reduce the fluctuations of x and thus improve the SNR by introducing a non-reciprocal coupling and driving the system out of equilibrium, while keeping the response of the system at all frequencies unaffected. Reducing the variance requires $\kappa \neq 0$, that is, just as for the reduction of the effective temperature, both reciprocal and non-reciprocal coupling are necessary to achieve enhanced sensing.

In practice, however, specifying the entire response function is often too restrictive, since we are rather interested in the response of the sensor at a specific frequency ω_0. We therefore specify the amplitude of the response $\bar{R}^X(\omega_0) = \bar{R}^X_{\mathrm{eq}}(\omega_0) \equiv R_0$, where

$$\bar{R}^X_{\mathrm{eq}}(\omega) = \sqrt{\frac{1}{k^2_{x,\mathrm{eq}} + (\gamma\omega)^2}} \tag{72}$$

is the response spectrum of the sensor in the absence of the demon. We remark that, in equilibrium, we have $\Xi^X_{\mathrm{eq}} = T/k_{x,\mathrm{eq}}$. Thus, a decrease in the fluctuations of X can only be achieved by increasing the force constant $k_{x,\mathrm{eq}}$, which, however, also decreases the response Eq. (72). Consequently, if we want to reduce the fluctuations while maintaining the response, we need to drive the system out of equilibrium. In the following, we therefore also specify the total rate of dissipation σ, which characterizes how far the overall system is out of equilibrium. Then, we minimize

the variance with respect to the eigenvalues λ^+ and λ^-, which gives us the least possible amount of fluctuations for a given response and dissipation. Since the corresponding optimization problem is non-linear with equality (on the response and dissipation) and inequality (on the eigenvalues, $\lambda^+, \lambda^- > 0$) constraints, we carry out the minimization numerically using the `NMinimize` command of Mathematica. Since at high frequencies, the response and fluctuations of the system are determined by the environment and we therefore cannot expect a significant enhancement of the response, we focus on the low-frequency limit $\omega_0 \to 0$. Numerically, we observe the scaling $\lambda^+ \propto 1/\sqrt{\omega_0}$ and $\lambda^- \propto \omega_0$ for the optimal eigenvalues in the limit $\omega_0 \to 0$. We therefore set

$$\lambda^+ = \frac{c_1}{\sqrt{\omega_0}} \qquad \text{and} \qquad \lambda^- = c_2\omega_0, \tag{73}$$

where c_1 and c_2 are positive constants. Plugging this into Eq. (70) and expanding for small ω_0, we obtain

$$\Xi^X \simeq \left(\frac{T}{c_1} + \frac{c_1(\gamma^2 + c_2^2)R_0^2 T}{c_2\gamma\sigma}\right)\sqrt{\omega_0} + O(\omega_0). \tag{74}$$

Minimizing the coefficient with respect to c_1 and c_2, we find the minimal fluctuations

$$\Xi^X \simeq 2\sqrt{2}TR_0\sqrt{\frac{\omega_0}{\sigma}} + O(\omega_0). \tag{75}$$

The covariance matrix is given by

$$\boldsymbol{\Xi} \simeq T\begin{pmatrix} 2R_0\sqrt{\frac{2\omega_0}{\sigma}} & \left(\frac{2R_0^2}{\gamma^2\sigma\omega_0}\right)^{\frac{1}{4}} \\ \left(\frac{2R_0^2}{\gamma^2\sigma\omega_0}\right)^{\frac{1}{4}} & \frac{1}{\gamma\omega_0}\end{pmatrix}. \tag{76}$$

We see that, for a low-frequency perturbation, the fluctuations of the sensor can be made arbitrarily small, vanishing as $\omega_0^{1/2}$, while maintaining a finite dissipation rate and a finite response to the perturbation. At the same time, the correlations between the sensor and the demon diverge as $\omega_0^{-1/4}$, while the fluctuations of the demon diverge even faster, as ω_0^{-1}. This is corroborated by the eigenvectors of the force matrix,

$$\boldsymbol{e}^- \simeq \begin{pmatrix} \left(\frac{2R_0^2\gamma^2\omega_0^3}{\sigma}\right)^{\frac{1}{4}} \\ 1 \end{pmatrix}, \qquad \boldsymbol{e}^+ \simeq \begin{pmatrix} -\left(\frac{1}{2R_0^2\gamma^2\sigma\omega_0}\right)^{\frac{1}{4}} \\ 1 \end{pmatrix}. \tag{77}$$

As the smaller eigenvalue λ^- vanishes, the corresponding eigenvector $\boldsymbol{e}^-$ is oriented in the direction of the demon; the dynamics of the demon become asymptotically unstable, leading to large fluctuations. By contrast, the larger eigenvalue λ^+ increases; its eigenvector is oriented in the direction of the sensor. The increase in λ^+ therefore stabilizes the sensor, decreasing its fluctuations.

While we defined the SNR Eq. (69) using the overall magnitude (absolute value) of the response, we can also consider the real and imaginary part of the response function separately. The real part $R^X(\omega)$, which is related to the imaginary part of the velocity response by $R^X(\omega) = \tilde{R}_v^X(\omega)/\omega$, measures the response of the sensor that is in phase with the input signal $\phi(t)$. Conversely, the imaginary part $R^X(\omega) = R_v^X(\omega)/\omega$ measures the out-of-phase component of the response. For the equilibrium system defined above, we have

$$R_{\text{eq}}^X(\omega) = \frac{k_{x,\text{eq}}}{k_{x,\text{eq}}^2 + (\gamma\omega)^2} \qquad \text{and} \qquad \tilde{R}_{\text{eq}}^X(\omega) = \frac{(\gamma\omega)}{k_{x,\text{eq}}^2 + (\gamma\omega)^2}, \tag{78}$$

so that only the in-phase component contributes in the low-frequency limit and the finite equilibrium SNR in the low-frequency limit originates from this component. The reason is that, for a sufficiently slow input signal, the sensor can follow the signal without any delay. We remark that, since the out-of-phase component corresponds to the real part of the velocity response entering the Harada-Sasa relation Eq. (42), the in-phase component is not directly related to the dissipation. In the presence of the demon and for the parameters maximizing the SNR, both the in-phase and out-of-phase response remain finite in the low-frequency limit. To leading order, we find

$$R^X(\omega_0) \simeq \tilde{R}^X(\omega_0) \simeq \frac{1}{\sqrt{2}k_{x,\text{eq}}} \simeq \frac{1}{\sqrt{2}}\bar{R}_{\text{eq}}^X(\omega_0), \tag{79}$$

where we recall that we optimized the parameters such that the response at ω_0 is equal to the equilibrium reference system, $\bar{R}^X(\omega_0) = \bar{R}_{\text{eq}}^X(\omega_0)$. Thus, in the presence of the demon, the output signal of the sensor acquires a phase-shift

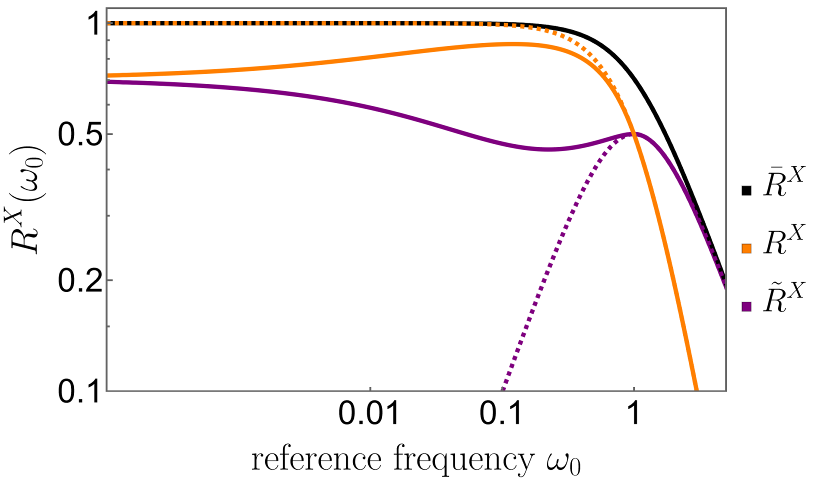

Supplementary Figure 1. The real (orange) and imaginary (purple) part, as well as the absolute value (black) of the response function as a function of the reference frequency ω_0. The solid lines correspond to the parameters maximizing the SNR in the presence of the demon for $\sigma = 1$ and $T = 1$. The dashed lines are the response function of the corresponding reference equilibrium system with the same absolute value of the response. While in the equilibrium system, the low-frequency response stems solely from the in-phase component, both the in- and out-of-phase component contribute equally to the response in the presence of the demon.

of $\frac{\pi}{4}$ with respect to the input signal. The fact that the out-of-phase component remains positive is responsible for the overall negative dissipation of the sensor via Eq. (45).

We can also consider the learning rate and rate of heat dissipation, which scale as

$$Q^X_{\mathrm{diss}} \simeq -Tl^Y \simeq -T\sqrt{\sigma\omega_0} + O(\omega_0), \qquad Q^Y_{\mathrm{diss}} \simeq T\sigma + T\sqrt{\sigma\omega_0} + O(\omega_0) \tag{80}$$

in the low-frequency limit. The entropy production rate associated with the sensor X scales as

$$\sigma^X \simeq 2\sqrt{2}\gamma R_0 \sqrt{\sigma}\omega_0^{\frac{3}{2}} + O(\omega_0^2). \tag{81}$$

Thus, the information acquired by the demon per period of the driving, l^Y/ω_0, diverges as $\omega_0^{-1/2}$ in the low-frequency limit; in order to suppress the fluctuations of the sensor, the demon has to learn about the fluctuations of the sensor and subsequently dissipate the acquired information in the form of heat into the environment. On the other hand, the dissipation from the sensor per period of the driving σ^X/ω_0 vanishes as $\omega_0^{1/2}$—in the low-frequency limit, the entire dissipation is due to the dynamics of the demon.

III. ENHANCED SNR IN A DISCRETE MODEL

In the main text and the above, we focused on enhanced sensing in continuous-space Langevin dynamics, specifically on a linear model involving two continuous degrees of freedom. This type of dynamics has the advantage that we have a direct relation between the fluctuations and response of the sensor, and the heat dissipation rate via the Harada-Sasa relation Eq. (45). While this relation provides a useful intuition, it is not required to achieve enhanced sensing out of equilibrium. Rather, the effect relies on the second law of information thermodynamics Eq. (13) together with non-reciprocal interactions and response theory. To demonstrate that enhanced sensing can be realized even in discrete-space systems, where no analog of the Harada-Sasa relation is known, we consider a system of two interacting classical spins, each of which can be in either the down ($\downarrow$, $s = -1$) or up ($\uparrow$, $s = 1$) state, see Fig. 2. Each of the spins can randomly flip; the rates of these spin-flips are determined by the overall energy of the system. We imagine that one spin, $s^X = \pm 1$, to correspond to the sensor, that is, we measure the change in the average orientation of the spin in response to a weak external field $\epsilon\phi(t)$ acting on this spin. The other spin $s^Y = \pm 1$ is an auxiliary system ("demon"), which through the interaction between the two spins affects both the response and fluctuations of the sensor spin. Similar to the continuous model discussed above, we allow for both reciprocal and non-reciprocal coupling, the magnitudes of these couplings are denoted by J and Δ respectively. The concrete transition rates are

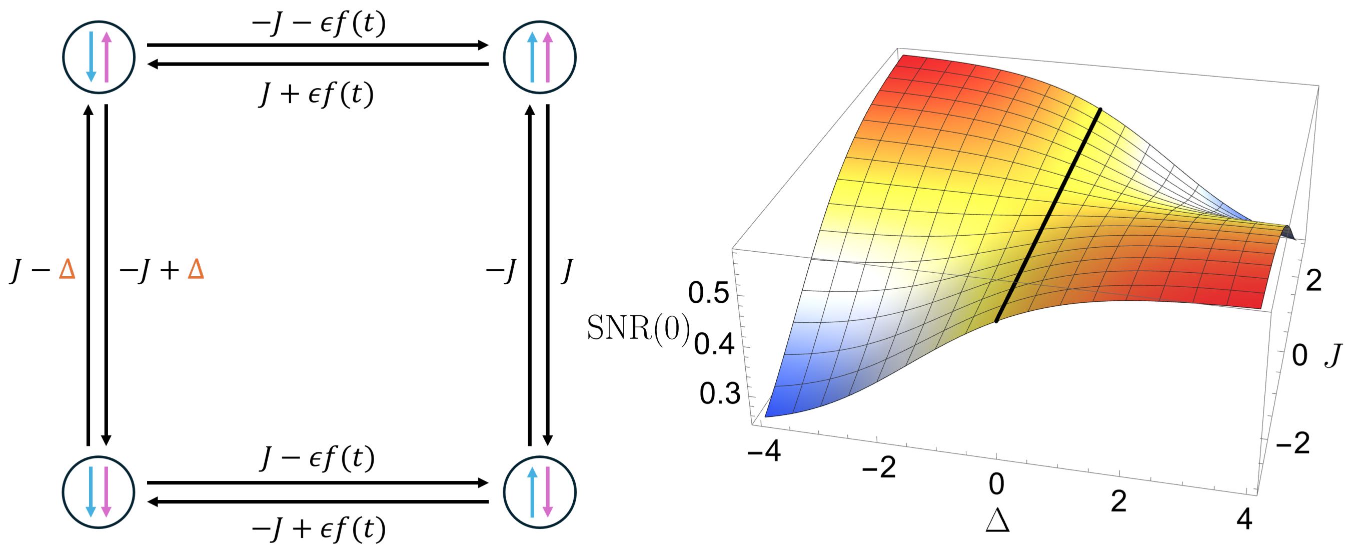

Supplementary Figure 2. (Left.) Illustration of a discrete spin model exhibiting increases SNR for non-reciprocal coupling. The sensor spin s^X (blue) and the demon spin s^Y (purple) interact via a reciprocal coupling of strength J; for positive J the coupling tends to align the spins, while for negative J, the coupling favors antiparallel orientation. The symbols next to the arrows indicate the change in total energy in the corresponding transition. In addition to the reciprocal coupling, we also introduce a non-reciprocal coupling of strength Δ; this coupling can be thought of applying an external field of magnitude Δ to the demon spin only if the sensor spin is in the down state. A weak, time-dependent perturbation $\epsilon\phi(t)$ acts on the sensor spin; we observe the corresponding change in the average orientation of the sensor spin.
(Right.) The SNR of the sensor spin at zero frequency as a function of the reciprocal and non-reciprocal coupling strength for $\beta = 0.5$. For only reciprocal coupling, the response of the sensor spin is unaffected by the coupling (black line). However, a combination of reciprocal and non-reciprocal coupling can increase the SNR beyond the equilibrium value (red-shaded areas).

given by

$$\begin{aligned} W_{(\downarrow\downarrow)\leftarrow(\uparrow\downarrow)} &= \frac{e^{\beta(J-\epsilon\phi(t))}}{e^{\beta(J-\epsilon\phi(t))}+e^{-\beta(J-\epsilon\phi(t))}}, \qquad & W_{(\uparrow\downarrow)\leftarrow(\downarrow\downarrow)} &= \frac{e^{-\beta(J-\epsilon\phi(t))}}{e^{\beta(J-\epsilon\phi(t))}+e^{-\beta(J-\epsilon\phi(t))}} \\ W_{(\uparrow\downarrow)\leftarrow(\uparrow\uparrow)} &= \frac{e^{\beta J}}{e^{\beta J}+e^{-\beta J}}, \qquad & W_{(\uparrow\uparrow)\leftarrow(\uparrow\downarrow)} &= \frac{e^{-\beta J}}{e^{\beta J}+e^{-\beta J}} \\ W_{(\downarrow\uparrow)\leftarrow(\uparrow\uparrow)} &= \frac{e^{\beta(J+\epsilon\phi(t))}}{e^{\beta(J+\epsilon\phi(t))}+e^{-\beta(J+\epsilon\phi(t))}}, \qquad & W_{(\uparrow\uparrow)\leftarrow(\downarrow\uparrow)} &= \frac{e^{-\beta(J+\epsilon\phi(t))}}{e^{\beta(J+\epsilon\phi(t))}+e^{-\beta(J+\epsilon\phi(t))}} \\ W_{(\downarrow\downarrow)\leftarrow(\downarrow\uparrow)} &= \frac{e^{\beta(J-\Delta)}}{e^{\beta(J-\Delta)}+e^{\beta(\Delta-J)}}, \qquad & W_{(\downarrow\uparrow)\leftarrow(\downarrow\downarrow)} &= \frac{e^{\beta(\Delta-J)}}{e^{\beta(J-\Delta)}+e^{\beta(\Delta-J)}}, \end{aligned} \tag{82}$$

where β is the inverse temperature. Note that we normalized the rates such that the forward and reverse rate across any transition always sum to unity. Changing from a parallel to an antiparallel configuration changes the energy by J and vice versa. In addition, when the sensor spin is in the down state, we imagine applying a field Δ to the demon spin; in other words, the energy changes by Δ when the demon flips from the up to the down state, but only if the sensor is in the down state. It can easily be verified that this type of interaction is non-reciprocal, since a cyclic sequence of transitions changes the energy by Δ (for a counter-clockwise cycle) or $-\Delta$ (for a clockwise cycle), which implies non-conservative forces. By contrast, the effect of the reciprocal coupling J and the perturbation $\epsilon\phi(t)$ along a cyclic transition sequence always cancels. For this system, the response function of the sensor spin is defined as

$$\partial_\epsilon \left\langle s^X(t) \right\rangle\big|_{\epsilon=0} = \int_0^t dt'\; \mathcal{R}^X(t-t')\phi(t'), \qquad R^X(\omega) = \int_0^\infty dt\; e^{i\omega t}\mathcal{R}^X(t), \tag{83}$$

and we define the SNR as

$$\mathrm{SNR}^X(\omega) = \frac{|R^X(\omega)|\phi}{\sqrt{\mathrm{Var}_{\mathrm{st}}(s^X)}}, \tag{84}$$

that is, the magnitude of the response divided by the magnitude of the fluctuations. This quantity is shown for $\omega = 0$ in the right panel of Fig. 2. For only reciprocal or non-reciprocal coupling, the SNR of the sensor is unaffected by the

presence of the demon spin. This is a consequence of the normalization of the rates Eq. (82), which ensures that an overall change in energy across any single transition does not affect the rates. Only when we apply both reciprocal and non-reciprocal coupling, the SNR of the sensor increases beyond its equilibrium value, demonstrating that enhanced sensing out of equilibrium can also be realized in discrete systems. However, a crucial difference between the discrete and continuous model is the magnitude of the fluctuations. On a discrete state space with a finite number of states, the fluctuations of any observable are bounded by the maximal variation of the observable,

$$\mathrm{Var}_{\mathrm{st}}(s^Y) \leq \frac{(s^Y_{\mathrm{max}} - s^Y_{\mathrm{min}})^2}{4} = 1. \tag{85}$$

This constrains the fluctuations of the demon, thus limiting its ability to act as a fluctuation sink for the sensor. Thus, as can be seen from Fig. 2, even at zero frequency, the maximal enhancement of the SNR is relatively modest. By contrast, on a continuous state space, the fluctuations of the demon can become arbitrarily large, allowing it to absorb an unlimited amount of fluctuations from the sensor and thus enhance the SNR arbitrarily. We conjecture that a similar effect can be observed in jump processes on a discrete state space as the number of states of the demon is increased.

---

[1] H. Risken, *The Fokker-Planck Equation* (Springer Berlin, 1986).

[2] U. Seifert, Stochastic thermodynamics, fluctuation theorems and molecular machines, Rep. Prog. Phys. **75**, 126001 (2012).

[3] J. M. Horowitz and M. Esposito, Thermodynamics with continuous information flow, Phys. Rev. X **4**, 031015 (2014).

[4] T. Speck and U. Seifert, Restoring a fluctuation-dissipation theorem in a nonequilibrium steady state, EPL (Europhys. Lett.) **74**, 391 (2006).

[5] T. Harada and S.-i. Sasa, Energy dissipation and violation of the fluctuation-response relation in nonequilibrium Langevin systems, Phys. Rev. E **73**, 026131 (2006).

[6] T. Harada and S.-i. Sasa, Equality connecting energy dissipation with a violation of the fluctuation-response relation, Phys. Rev. Lett. **95**, 130602 (2005).

[7] L. F. Cugliandolo, The effective temperature, J. Phys. A: Math. Theor. **44**, 483001 (2011).
